# Performing Isometric Force Control in Combination with a Cognitive Task: A Multidimensional Assessment

**DOI:** 10.1371/journal.pone.0142627

**Published:** 2015-11-16

**Authors:** Jean-Jacques Temprado, Solveig Vieluf, Nicolas Bricot, Eric Berton, Rita Sleimen-Malkoun

**Affiliations:** 1 Aix-Marseille Université, CNRS, Institut des Sciences du Mouvement UMR 7287, Marseille, France; 2 Collège Ostéopathique de Provence, Aix-Marseille, Marseille, France; 3 Aix-Marseille Université, Inserm, Institut de Neurosciences des Systèmes UMR_S 1106, Marseille, France; Beijing University of Posts and Telecommunications, CHINA

## Abstract

**Introduction:**

We used a multidimensional approach to study isometric force control in single and dual-task conditions.

**Methods:**

Multiple measures of performance, efficiency, variability, and structural interference were calculated at low and higher force levels under single (force maintenance) and dual-task (force maintenance and reaction time) conditions.

**Results:**

Reaction time and signal-to-noise ratio were larger in the dual-task conditions. They were also greater for the higher force condition, while sample entropy was lower. Perturbation analyses revealed smaller relative amplitude of downward perturbations for the higher force level.

**Discussion:**

Attentional effort and efficiency are positively related when force level increases, and inversely related to entropy. These relations were presumably mediated by attentional investment. Behavioral perturbations show that attentional resources and structural interference models are not mutually exclusive to account for dual-task situation. Overall, the present study highlights the interest of a multidimensional assessment of force control.

## Introduction

In isometric force control tasks, participants are requested to produce and maintain a given level of force as instructed by a visually presented target line [1–6]. In these situations, changes in the underlying organization of the neuro-musculo-behavioral system, which are associated with increases in the produced force level, are currently inferred from measures of: i) stability–e.g., standard deviation [3,7], ii) information processing efficiency–e.g., signal-to-noise ratio [1,3], and iii) complexity, through the correlated structure of variability of force outputs over one or multiple time scales–e.g., single or multi-scale entropy measures [1,3]. These metrics have been used separately to assess behavioral performance in most studies using force control tasks. Nevertheless, it has been shown that combining stability, efficiency, and complexity measures draw a more precise picture of how task demands, i.e., force level, affect the functioning of the system [1,3].

Another aspect of the functional organization of the force control system that has been scarcely investigated concerns the potential contribution of central processes, especially at higher force levels. Presumably, producing a higher force level increases the attentional effort [6]. However, the question remains of how central demands relate to information processing efficiency and complexity measures. The present study addressed this issue by using a dual-task paradigm, where two different force levels must be produced and maintained while concurrently performing a discrete cognitive task.

The dual-task paradigm has been widely used to investigate the association of continuous motor tasks (e.g., bimanual coordination, posture, locomotion…) and discrete probe reaction time tasks [8–14]. In these situations, the amount of central resources needed to achieve the primary task is usually assessed through the increase in reaction time, relative to when the reaction time is performed alone. Specifically, an increase in reaction time is interpreted in terms of fixed-capacity model of attention: the more central resources are engaged in the primary task, the larger the increase in reaction time is in the dual-task situation. Few studies supported the hypothesis of increased central demands over force levels [6,15–17]. For instance, when comparing two different levels of relative force (30 and 60% of the individual’s maximum voluntary contraction (MVC)), Zijdewind et al. [6] observed an increase in RT at the higher force level condition. They suggested that increasing force level induced a change in central drive of the peripheral muscular system, reflected by increased activity and excitability of the dorsolateral prefrontal motor cortex, which is known to be involved in executive functioning [18]. These changes presumably explain the stronger demands on the attentional system.

While the resource allocation model provides a plausible explanation for attention sharing in dual-task situations, another mechanism, so-called structural interference, has also proven to play an important role in dual-task situations [19,20]. Structural interference is hypothesized to result from the outcome conflict that arises when activity in central structures dedicated to one task (e.g., RT) produces harmful effects (overflow) on structures dedicated to the other concomitant task. At the behavioral level, structural interference leads to perturbations of outputs–a momentarily halt of the primary task or change in the output value—as the result of simultaneously performing the reaction time task [19,20].

Thus, attentional resource allocation and structural interference reflect two different facets of the dual-task situations, yet, they are often considered as mutually exclusive in the literature [6,20]. However, in a previous study, Temprado et al. [19] have shown that they can be reconciled. Specifically, by combining a bimanual coordination task and a reaction time task (performed with the feet), they found that the amplitude of perturbations was closely related to the stability and efficiency of the bimanual coordination system: the less stable/efficient the bimanual coordination, the larger the amplitude of perturbation. In addition, since behavioral stability/efficiency strongly depended on the gradual allocation/withdrawal of attentional resources, perturbations resulting from structural interference were also related to central costs: the more the attentional resources allocated to the primary task, the higher the reaction time and the smaller the perturbation [19]. To our knowledge, a multidimensional analysis of how the attentional demands, structural interference, stability, efficiency and complexity of the force control system relate to each other when force level increases has never been conducted before.

In the present study, we applied this framework to investigate behavioral perturbations resulting from the association of an isometric force control task (performed with the right index finger) and a reaction time task (performed with the left foot). Specifically, we compared two levels of relative force (10 and 50% of MVC) performed in single and dual-task situations to explore how attentional demands and structural interference: i) changed with increasing force level, and ii) were related to variability, signal-to-noise ratio and entropy measures of the observed performance.

Different hypotheses were tested. First of all, we predicted that the dual-task situation would force the participants to release a part of their attentional resources from the force control task. As a consequence, higher variability and lower efficiency should be observed in the dual-task conditions relative to single task conditions. These detrimental effects should be accompanied by higher entropy of force fluctuations (18–21). Secondly, increasing force level (from 10 to 50% of MVC) was predicted to require more attentional effort. Thus, we expected, on the one hand, a longer reaction time for the higher force level [6], and on the other hand, a higher signal-to-noise ratio (efficiency) and a larger entropy value (higher complexity) [1,3]. Thirdly, we predicted to observe structural interference between the reaction time and the force control tasks. In this respect, our main hypothesis was that increasing force level should lead to more interference with the foot reaction time task, since more common central components are involved [6]. Consequently, behavioral perturbations should be larger at the higher force level. However, according to the results of previous studies [12,19], prioritization strategy could be different between low and higher force levels (i.e., increase in prioritization at the higher force level). In this case, the hypothesis would be that behavioral perturbations should be smaller with increasing force level since more attention is needed to control the produced force. This would modify the coupling between the functional components of the force control system, and thereby, attenuate the behavioral consequences of central interference [19].

## Methods

### Participants

25 right-handed students (mean age: 23.44±1.55 years; 12 women) gave their informed consent to voluntarily participate in the study. Procedures were approved by the local ethic committee of the Aix-Marseille University and were in accordance with the ethical standards laid down in the Declaration of Helsinki.

### Experimental setup

**Fig 1:**
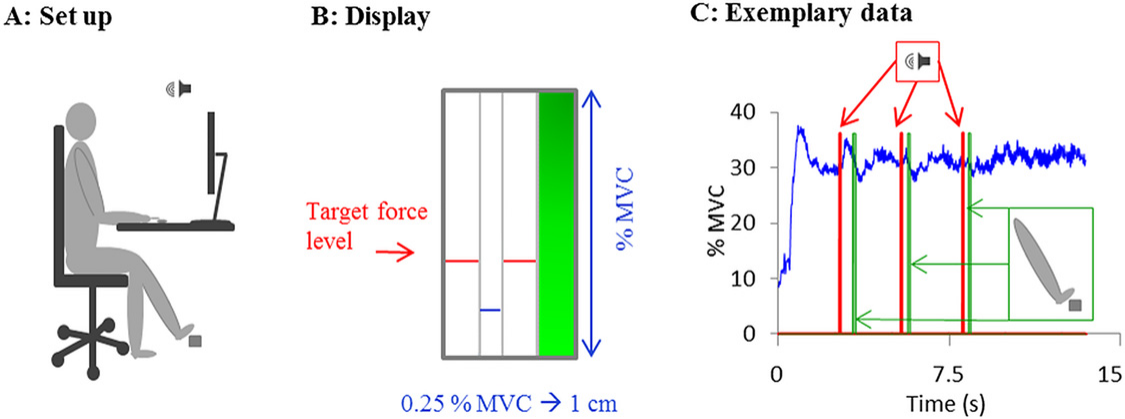
Experimental setup. A: Experimental setup. Participants were seated with their right index finger and their left foot reaching comfortably the force transducer and the response button, respectively. Auditory stimuli were provided via loudspeakers. B: the visual feedback that was provided on the screen representing the applied force (blue line) and the target force level (red lines). C: An example of a time-series showing perturbations from the dual task condition. Participants were instructed to maintain a given force level (applied force: blue line) and to react (green line) as quickly as possible when hearing the auditory beep (red line).

Participants were seated at a table facing a 19” screen. The arms were comfortably positioned in a way that the right index finger was placed on a force transducer (SCAIME, ZFA, 50 kg) and the left foot was placed on a response button (Fig 1A). A customized LabView (National Instruments, Austin, TX) program and a National Instruments acquisition card (DAQ NI-USB 6008- National Instruments) were used to collect the force and reaction time data, to provide visual feedback, as well as auditory and visual stimuli to the participants. The participants’ task was to maintain a certain force level by pressing the transducer with their right index finger (single force maintenance task). In dual-task conditions, the force maintenance task was combined with a reaction time task (RTT) in which participants had to react as fast as possible to a given tone by pressing the response button with their left foot. The RTT was also performed separately as single reaction time task (SRTT). Commonly for all tasks the start and the duration of the trials were indicated to the participant on the screen by a bar next to the target area that was illuminated throughout the trial (Fig 1B). For the single reaction time task, the trials started automatically after a break of 5 s and auditory signals were presented via the computers loud speaker. In both single and dual-task conditions, each force trial was initiated by the participants when starting to press the force transducer. Data collection began as soon as the average value of 20 sample points exceeded a threshold of 2 N. The participants were instructed to release their force when the bar turned off. An exemplary trial can be seen in Fig 1C. The start of each trial was self-paced, but a mandatory break of 5 s was implemented in the program. The target force level to be maintained was indicated on the screen with two red lines (2 points width), between which a blue line could be moved up and down as function of the force applied on the force transducer. The participants were instructed to align the blue line with the red ones (Fig 1B). Data were sampled at 240 Hz and saved for later analysis.

### Tasks and procedure

Participants were informed verbally about the general procedure and had to undergo several familiarization steps. After a general familiarization with the device and the display, MVC was determined for each participant. In line with previous studies [1–5], MVC was determined as average of the mean force produced during the last 3 s of each trial, and was used to calculate relative target force levels of the consecutive force control tasks. A resting period of 30 s was given between trials. As a familiarization with the force control tasks, 2 trials at 15 and 45% of the MVC were presented randomly. After that, the single task conditions at 10 and 50% of MVC were presented in random order. 6 trials of 15 s of the 10 and 50% force levels were randomly presented. 10 reaction times were performed to familiarize with the device, followed by the single reaction time task containing 6 trials of 15 s duration with 3 auditory stimuli, with a similar setup and timing as in the dual task conditions. The first tone occurred after 3 s then the inter-stimulus-interval was randomized between 3 and 6 s. Two trials were performed by each participant to familiarize with the dual-task before it was recorded.

### Data analysis

**Fig 2:**
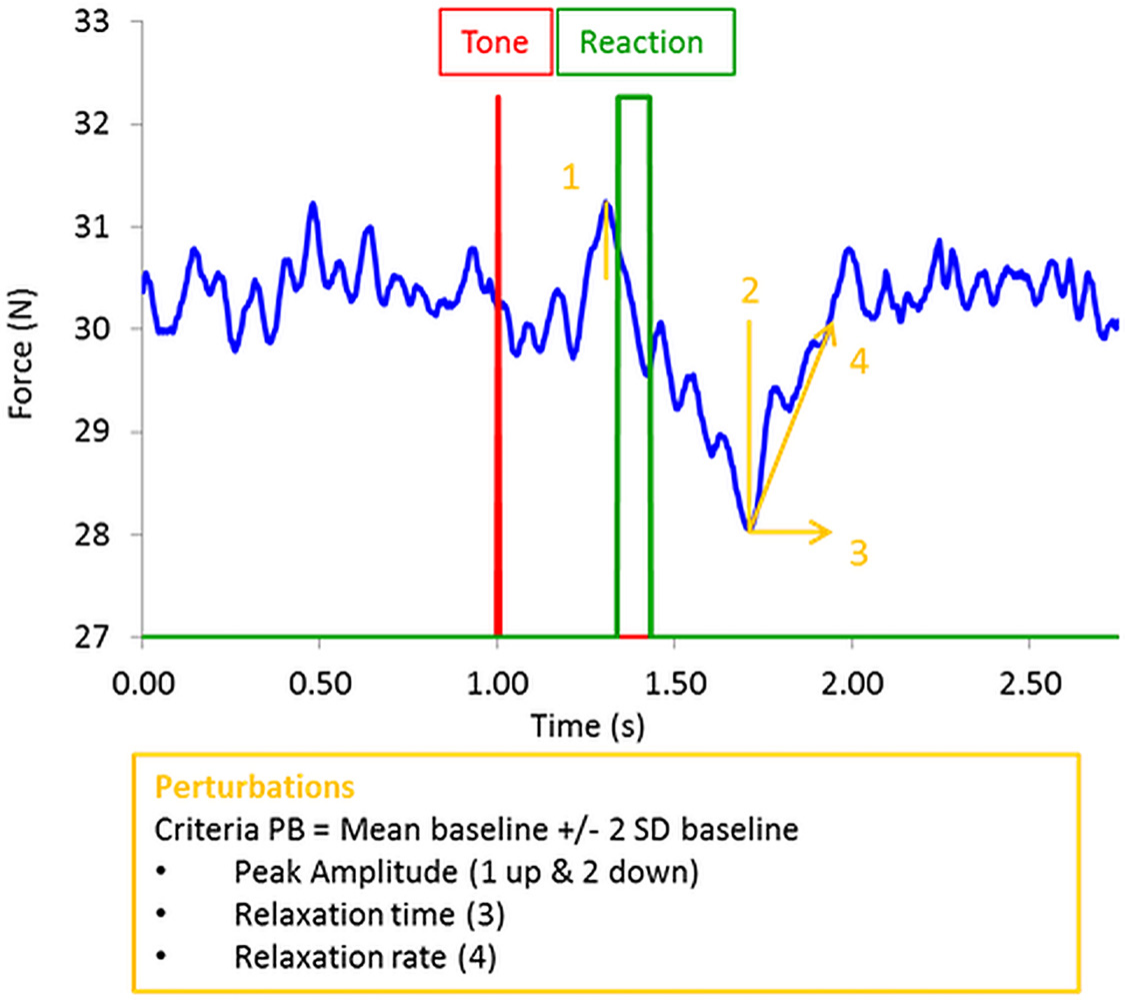
Force-time-series. Representative force-time-series showing a typical perturbation profile. The criterion for the perturbation detection was defined by values crossing the mean of a 1 s baseline before the tone ±2 SD of the baseline. Based on this criterion, first, the perturbation upward is detected and expressed in amplitude relative to the prescribed force level; second, the downward perturbation is characterized in the same way. Based on the amplitude and the relaxation time, i.e., the time from the peak amplitude of the downward perturbation till the force reaches the perturbation criteria again, we calculated the rate of relaxation.

On the basis of all 18 reactions per force level, the reaction times were trimmed: the 3 (17%) fastest and 3 (17%) slowest reactions were excluded from further analysis, and then remaining values were averaged [6]. The acquired data of the produced force over time were analyzed with the use of a Matlab R2012b customized program (MathWorks, Natick, MA, USA). Data were converted to relative force values in percentage of the participants’ individual MVC and low-pass filtered with a 4th order Butterworth filter at 30 Hz. For the single task, the first 3 s were discarded from the trial and then the trial was cut in segments of 3 s. To analyze a stable period of force maintenance under dual-task conditions, the last 3 s of the 6 s intervals were taken into account. All variables were calculated per segment and then averaged per participant. To characterize general properties of force production, the mean force and its SD were calculated for each trial of each participant and in each force condition. As a measure of efficiency, the signal-to-noise ratio was determined by dividing the mean force by the SD. To characterize the variability structure of the time series, a measure of the signal’s irregularity, sample entropy (SampEn; [21]), was calculated, with higher SampEn values reflecting higher irregularity. Two types of perturbations around the period of the reaction were detected in the dual task conditions: an upward (i.e., leading to an increase in the force produced) and a downward perturbation (i.e., leading to a decrease in the force produced) (Fig 2). The criterion for detecting a perturbation and also for the return to the initial force level was the mean force applied during the 1 s before the tone ±2 SD calculated at the baseline. We analyzed: i) the amplitude of both perturbations in percentage of the mean force applied per trial at the respective force level, ii) total relaxation time that is, the time to recover the perturbation and get to the initial force level, and iii) rate of relaxation that is, the amount of force recovered per unit of time expressed in % of MVC per ms. To compare the impact that the perturbation period has on the general characteristics of force control, we compared the values of the mean, SD, signal-to-noise ratio and SampEn, for the 1 s period before each tone occurred with those of the 1 s after each relaxation of the system.

Statistical analyses were conducted in STATISTICA (StatSoft, Tulsa, OK, USA). 2 Task condition (single, dual) × 2 Force level (10%, 50%), or a 2 Time (before, after) × 2 Force level (10%, 50%) repeated measure ANOVA were performed to test for statistical significance. For the reaction time the two force levels were compared with the single task by a repeated measure ANOVA and similarly the perturbation amplitude, relaxation time, and rate of relaxation were compared for the two force levels. As a prerequisite we included gender as a variable and age and MVC as covariates. However, theses variables did not have a significant influence on the results. Therefore, they were not reported. The sphericity of the data was verified with the test of Mauchley. The Greenhouse-Geisser correction was applied when necessary. The level of significance was set to p<0.05. Effect sizes are given as partial Eta squares (η_p_^2^). Significant interaction effects were followed by Newman-Keuls’ post-hoc test.

## Results

A summary of the general properties of force production, perturbation characteristics, and the mean performance in the reaction time task are presented in Table 1.

**Table 1:** General properties of force production.

		Mean	SD	S-t-N	SampEn
Single task	10%	9,936 (0,213)	0,363 (0,083)	28,662 (6,278)	0,771 (0,217)
	50%	48,664 (0,672)	1,138 (0355)	46,354 (12,667)	0,410 (0,122)
Dual task	10%	9,969 (0,365)	0,376 (0,196)	30,770 (8,786)	0,738 (0,177)
	50%	47,963 (1,980)	1,331 (0,526)	41,880 (15,339)	0,411 (0,106)
		Amplitude up	Amplitude down	Relaxation time	Rate of relaxation
Perturbation	10%	6,707 (3,212)	10,885 (3,440)	366,456 (80,863)	0,0026 (0,0013)
	50%	5,728 (2,431)	8,672 (2,802)	465,467 (77,055)	0,0133 (0,0039)
		Mean	SD	S-t-N	SampEn
Before	10%	10,033 (0,274)	0,307 (0,069)	40,587 (10,490)	0,938 (0,231)
	50%	48,277 (1,032)	1,198 (0,359)	52,192 (16,076)	0,438 (0,218)
After	10%	10,206 (0,466)	0,740 (0,195)	17,660 (4,675)	0,426 (0,37)
	50%	44,031 (2,452)	3,932 (1,105)	15,845 (5,601)	0,143 (0,054)
		Single task	Dual-task 10%	Dual-task 50%	
Reaction time		263,643 (31,057)	279,262 (31,276)	290,290 (36,112)	

Means and standard deviations of the general properties of force production (mean, SD, signal-to-noise ratio (S-t-N), and SampEn) as well as perturbation characteristics (relative amplitude, relaxation time, and relaxation rate), and reaction time averaged per group and condition.

### Comparison of single and dual tasks

#### Mean force level

**Fig 3:**
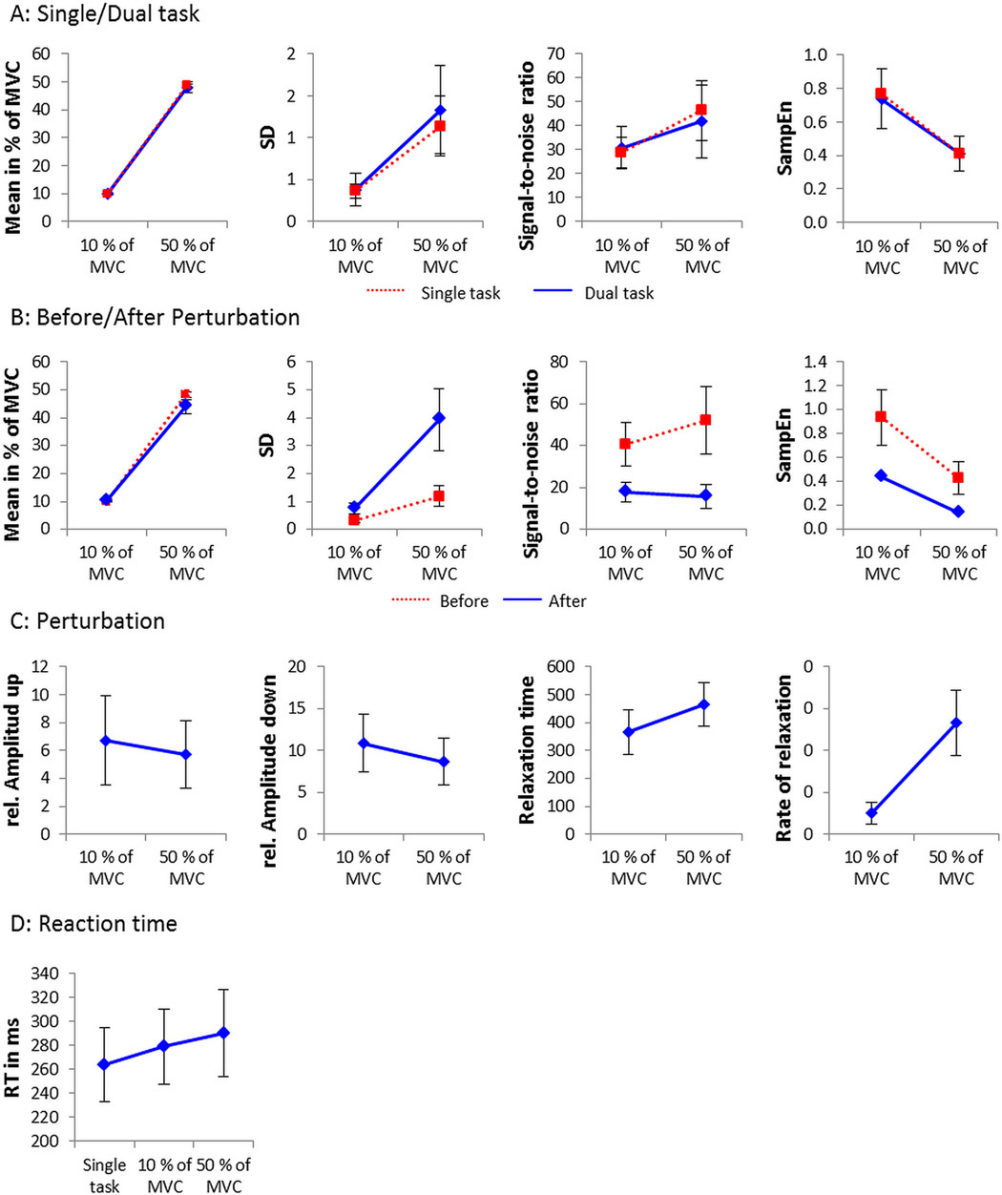
General properties of force production. A: Mean force, standard deviation, signal-to-noise ratio, and SampEn as a function of force level plotted per group for the single and dual task comparison. B: the comparison before and after the perturbation. C: the relative amplitude of the up- and the downward perturbation, the relaxation time, and the rate of relaxation. Additionally reaction times were plotted as a function of force level. Each data point represents a group mean. Error bars represent the standard deviation.

We compared mean relative force levels between the two conditions (10 and 50%), in the two tasks (ST and DT) (Fig 3A, first panel). The analysis showed that the conditions were significantly different, *F*(1, 24) = 29986.33, p < .001, η_p_^2^ = .999 (9.97 and 47.9%, respectively). The task × force level interaction was marginally significant, *F*(1,24) = 3.50, *p* = .07, η_p_^2^ = .127. No difference was found between single and dual tasks, *F*(1,24) = 2.59, *p* = .12, η_p_^2^ = .098.

#### Standard deviation (SD)

The SDs observed in the two conditions were significantly different *F*(1,24) = 164.98, p < .001, η_p_^2^ = .873 (0.38% and 1.34%, respectively). A significant task × force level interaction effect was also observed, *F*(1,24) = 4.30, *p* = .05, η_p_^2^ = .152 (Fig 3A, second panel). Post-hoc decomposition showed SD increased with force level in both the ST and DT. However, SD was larger in the DT condition only for the higher force level (50%). The main effect of task was marginally significant, *F*(1,24) = 3.07, *p* = .09, η_p_^2^ = .113.

#### Signal-to-noise ratio

The analysis showed that the signal-to-noise ratio was larger in the 50% condition (41.7) than in the 10% condition (30.5), *F*(1,24) = 39.94, *p* < .01, η_p_^2^ = .625. A significant task × force level interaction effect was also observed, *F*(1,24) = 5.01, *p* = .04, η_p_^2^ = .173 (Fig 3A, third panel). Post-hoc decomposition showed signal-to-noise ratio increased with force level in both the ST and DT. However, signal-to-noise ratio was larger in the DT condition only for the higher force level (50%). The main effect of task was not significant, *F*(1,24) = 0.57, *p* = .46, η_p_^2^ = .023.

#### Sample entropy (SampEn)

The analysis of the regularity of the signal by means of SampEn showed that the difference between 10 and 50% was significant: the complexity decreased in the 50% condition relative to the 10% condition, *F*(1,24) = 116.95, p < .01, η_p_^2^ = .830. No task by force level interaction, *F*(1,24) = 0.61, *p* = .44, η_p_^2^ = .025, and no difference between single and dual task, *F*(1,24) = 0.43, *p* = .52, η_p_^2^ = .017, was observed (Fig 3A, forth panel).

#### Reaction time

In the dual-task conditions, post-hoc comparison of the significant force level/task effect, *F*(1,24) = 19.42, p < .01, η_p_^2^ = .447, confirmed that the mean reaction time was higher in the 50% condition (290.3 ms) than in the 10% condition (279.3 ms) and both were higher than the reaction time during the single task (263.6 ms) (Fig 3D).

### Perturbation analysis in dual-task conditions

#### Amplitude of perturbations

We analyzed the relative amplitude of upward and downward perturbations (Fig 3C, first two panels). The analysis showed that the downward perturbation was smaller in the 50% condition (8.7%) than in the 10% condition (11.0%), *F*(1,24) = 10.86, *p* < .01, η_p_^2^ = .311. No difference was found between 10 and 50% for the upward perturbation (6.7% and 5.7%, respectively), *F*(1,24) = 1.82, *p* = .19, η_p_^2^ = .073.

#### Relaxation time

The analysis revealed a significant force level effect, *F*(1,24) = 28.49, *p* < .01, η_p_^2^ = .543. The relaxation time was shorter for the 10% (366.5 ms) than for the 50% (465.5 ms) condition (Fig 3C, third panel).

#### Rate of relaxation

The relaxation rate expresses the increase of force over time. Analysis showed that the rate of relaxation is higher for the 50% (0.01334% of MVC/ms) than for the 10% (0.0026% of MVC/ms), *F*(1,24) = 265.05, *p* < .01, η_p_^2^ = .917, indicating faster recovery in relative terms (Fig 3C, forth panel).

### Comparison of the period before and after the perturbation

#### Mean force level

The analysis of the mean force level before and after the perturbation showed, besides that the force level was higher for the 50% than for the 10%, *F*(1,24) = 13502.56, *p* < .01, η_p_^2^ = .998, that it was higher before than after the perturbation, *F*(1,24) = 82.11, *p* < .01, η_p_^2^ = .774. A significant time by force level interaction was revealed, *F*(1,24) = 104.42, *p* < .01, η_p_^2^ = .813 (Fig 3B, first panel). Post-hoc decomposition showed a significant difference for the comparison of before and after the perturbation for the 50% but not for the 10%.

#### Standard deviation (SD)

The SD was higher for 50% than for 10%, *F*(1,24) = 236.99, *p* < .01, η_p_^2^ = .908, and higher after than before the perturbation, *F*(1,24) = 256.74, *p* < .01, η_p_^2^ = .915. A significant time by force level interaction, *F*(1,24) = 120.87, *p* < .01, η_p_^2^ = .834, with all post-hoc comparisons showing significance, was observed (Fig 3B, second panel).

#### Signal-to-noise ratio

For the signal-to-noise ratio main effects of force level, *F*(1,24) = 5.29, *p* = .03, η_p_^2^ = .181, and time, *F*(1,24) = 179.59, *p* < .00, η_p_^2^ = .882, were revealed. The post-hoc comparison of the significant interaction, *F*(1,24) = 34.79, *p* < .00, η_p_^2^ = .592, showed that before the perturbation the signal-to-noise ratio was higher for the 50% than for the 10%, whereas after the perturbation no difference between force levels was revealed (Fig 3B, third panel).

#### Sample entropy (SampEn)

Analysis of the SampEn revealed a significant interaction of force level and time, *F*(1,24) = 34.78, *p* < .00, η_p_^2^ = .592 (Fig 3B, forth panel). Post-hoc comparison showed significant differences between force levels before and after the perturbation, however, the difference is higher before than after. Further, main effects of force level, *F*(1,24) = 136.93, *p* < .01, η_p_^2^ = .851, and time, *F*(1,24) = 212.26, *p* < .01, η_p_^2^ = .898, were significant.

## Discussion

In the present study, we used a dual-task paradigm to investigate how attentional demands, structural interference, stability, efficiency, and complexity of the force control system relate to each other at two different force levels.

### Comparison between single and dual-tasks: effect of releasing attentional resources

By comparing the single and dual-tasks, we aimed to assess the effects of releasing attentional resources from force control on the stability, efficiency, and complexity of behavioral outputs. The results show higher variability and lower efficiency in the dual-task conditions, relative to the single task conditions. Thus, releasing attentional effort was detrimental for force control performance. However, it did not affect the temporal structure, i.e., complexity, of force signal. This result could suggest that the participants prioritized the force control task and invested a high level of attentional effort in the dual-task conditions. However, such a hypothesis is not comforted by the observed differences in terms of reaction times, variability, and efficiency between the single and dual-task conditions, which suggest that the participants’ attention was diverted, at least partly, from the primary force control task. Since a discrete reaction time was used as secondary task, an alternative explanation could be that the release of attentional resources was temporary that is, more pronounced just before the predicted occurrence of the response signal. This interpretation corroborate with the observed increase in SampEn in dual-task studies on postural control [22–24], which involved continuous cognitive tasks resulting in a more lasting diversion of attentional resources over the trial duration.

### Multidimensional comparison between force levels in the dual-task situation

By comparing two different force levels, we aimed at better understanding how stability, efficiency, complexity, and central demands evolved when specific task constraints are varied. The present results show a higher efficiency (signal-to-noise ratio) for the 50% condition, which is consistent with previous findings [1,3]. Attentional demands, quantified through RT, also increased with force level, which suggests that, in addition to peripheral factors, central processes also contribute to isometric force production. These results confirm those reported by Zijdewind et al. [6] for higher force levels (30 and 60%) by combining an isometric force maintenance task with a more complex reaction time task. Extending these finding to lower force levels, which are frequently used in daily living tasks and are known to be very sensitive to organismic constraints (e.g., aging), is of interest. It is also the case for showing that attentional demands related to isometric force control can be assessed through the use of a simple reaction time task. Indeed, few studies reported facilitating effects of the dual-task situation on force control processes when a simple reaction time task is used [6]. Though speculative due to the lack of direct measures of brain activity, the increase in reaction time associated with the higher force level can be interpreted as evidence of stronger demands on the attentional system that is, on brain areas and neural circuits related to the cognitive functions that are involved in both the force maintenance and the reaction time tasks [6,18]. Indeed, it is unlikely that the increase in reaction time could be explained by the effect of fatigue, as it was controlled for through the choice of force levels and trials duration, as well as by the verification of individual MVC values, which did not change after the completion of the experiment.

The combination of efficiency and complexity measures (i.e., signal-to-noise ratio and entropy, respectively) with those of attentional demands afforded a global picture of the coherent organization of the force control system. Our results show that the increase in the signal-to-noise ratio was associated with a decrease in entropy values. At least, this inverse relation suggests that changes in the internal organization of the force control system resulting from increasing force level (e.g., central demands motor units recruitment, types of fibers, coordination between muscle activations…) are reflected by a decrease in complexity of force fluctuations. These changes were associated with more efficient processing of information. A plausible interpretation is that the attentional effort, which was devoted to maintain the higher force level, mediated the relation between efficiency and complexity. This hypothesis is supported by previous studies on postural control [22,24], where it was shown that the increase in attentional demands is currently associated with a decrease in the entropy of the fluctuations of the center of pressure. The present study extends these results to force fluctuations in an isometric force control task. How the attentional effort acts on the different mechanisms involved in force control is still unknown. One can hypothesize that the attentional effort modifies the interactions between the different functional mechanisms by increasing their mutual couplings. Indeed, it has been suggested that the irregularity of fluctuations (i.e., higher entropy) reflects the degree of isolation of the different components in the system under scrutiny [25]. Thus, the force-related decrease in SampEn is suggestive of a reinforcement of mutual couplings between the functional components of the force control system, presumably as a result of increased attentional effort [9,19]. However, it is also plausible that, in addition to common demands on the attentional system, the two tasks generated interference between neural structures/motor mechanisms that are needed to perform the two tasks. In other words, with increasing force level, more common brain structures might be involved in the production of the finger and the foot responses. To address this issue, we analyzed the behavioral perturbations that resulted from foot response.

### Structural interference between the force control and the reaction time tasks

Two alternative predictions were tested through the analysis of behavioral perturbations. On the one hand, perturbations would be larger with increasing force level since more common central motor components are involved between the two tasks [6]. On the other hand, behavioral perturbations would remain unchanged or get even smaller at the higher force level, since the required extra-attentional effort would attenuate the behavioral consequences of central interference between the two tasks [19].

First of all, our results show the existence of two types of behavioral perturbations–namely, upward (increase in force) and downward (decrease in force) perturbations, which reflected structural interference between the two tasks [19,20]. Presumably, upward perturbations resulted from a directional coupling between foot movement and the direction of the isometric force applied to the sensor [26,27]. In support of this interpretation, it must be noted that the temporal occurrence is related to the reaction time as it occurs continuously slightly before or at the moment of the reaction (see Fig 2). Downward perturbations can be interpreted as a result of the silent, refractory period in the system, which leads to loss of force control and a release of the force applied on the sensor. Strikingly, perturbation analysis shows (for the first time to our knowledge) that structural interference strongly altered the force control system, as indicated by the changes observed in the signal-to-noise ratio 1 s after the perturbation that was induced by the foot response. Specifically, as a result of structural interference, efficiency dramatically decreased and the difference between force levels disappeared. Perturbation analysis also shows that: i) the upward perturbation remained unchanged over the two force levels, and ii) the downward perturbations decreased (when expressed in relation to the relative force level) at the higher force level. Accordingly, on the one hand, the comparison of pre- and post-perturbation periods suggests that structural interference was more detrimental for the higher force level. On the other hand, results observed for the relative amplitude of downward perturbation do not support the hypothesis of an increase in interference between common motor structures in the brain with force level. Conversely, this result rather suggests that higher attentional effort was associated with an attenuation of the downward perturbation and an increase in the rate of relaxation. Consistent results have been reported for the perturbations of relative phase in a bimanual coordination task [19]. Accordingly, a plausible interpretation is that, by reinforcing the coupling between functional components involved in force control, attentional effort: i) makes the force control system more resistant to central interference, and ii) contributes to a quicker recovery after the perturbation. The comparison of entropy measured before and after the perturbation does lend credence to this hypothesis. Indeed, entropy of force fluctuations decreased for both force level during the stabilized period following the perturbation. This result suggests an increase in the attentional effort dedicated to recover and maintain the initial force level after the perturbation.

## Conclusion and Perspectives

The present study provides a novel multidimensional assessment of force control through the combination of signal-to-noise ratio, sample entropy, and reaction time metrics. In particular, it contributes to a better understanding of how central demands associated with the maintenance of different levels of isometric force are related to efficiency and complexity of the force control system.

Our study demonstrates and extends the observations that were made by Zijdewind et al. [6] regarding the increase of attentional load with increasing force requirements. It also shows that efficiency and attentional demands are positively related: the higher the attentional effort, the higher the efficiency. Moreover, entropy of force fluctuations is inversely related to attentional demands. Our results extend to force control those observed in previous studies on postural control. Analysis of behavioral perturbations shows that these perturbations are attenuated for the higher force level. It strongly suggests that the structural interference that occurs as a result of combining force control and reaction tasks cannot explain alone the increase in reaction time observed for the higher force level. In addition, the comparison of the signal-to-noise ratio and entropy values pre- and post-perturbation reveals how the force control system accommodates the interference to relax toward its initial state. Thus, taken together, the results of the present study confirm that attentional resource and structural interference/outcome conflict models are not mutually exclusive to account for dual-task situations [19].

## References

[1] Vieluf S, Temprado J, Berton E, Jirsa VK, Sleimen-Malkoun R. Effects of task and age on the magnitude and structure of force fluctuations: insights into underlying neuro-behavioral processes. BMC Neurosci. 2015;16: 12 10.1186/s12868-015-0153-7 25887599 PMC4359767

[2] Lindberg P, Ody C, Feydy A, Maier MA. Precision in isometric precision grip force is reduced in middle-aged adults. Exp Brain Res. 2009;193: 213–224. 10.1007/s00221-008-1613-4 18953529

[3] Slifkin AB, Newell KM. Noise, information transmission, and force variability. J Exp Psychol Human. 1999;25: 837–851. 10.1037//0096-1523.25.3.837 10385989

[4] Voelcker-Rehage C, Alberts JL. Effect of motor practice on dual-task performance in older adults. J Gerontol B Psychol Sci Soc Sci. 2007;62: P141–P148. 17507581 10.1093/geronb/62.3.p141

[5] Sosnoff JJ, Newell KM. Are age-related increases in force variability due to decrements in strength? Exp Brain Res. 2006;174: 86–94. 16575579 10.1007/s00221-006-0422-x

[6] Zijdewind I, van Duinen H, Zielman R, Lorist MM. Interaction between force production and cognitive performance in humans. Clin Neurophysiol. 2006;117: 660–667. 16434230 10.1016/j.clinph.2005.11.016

[7] Christou EA, Carlton LG. Age and contraction type influence motor output variability in rapid discrete tasks. J Appl Physiol (1985). 2002;93: 489–498. 12133855 10.1152/japplphysiol.00335.2001

[8] Pellecchia GL, Shockley K, Turvey MT. Concurrent cognitive task modulates coordination dynamics. Cognitive Sci. 2005;29: 531–557. 10.1207/s15516709cog0000_12 21702784

[9] Temprado J, Zanone P, Monno A, Laurent M. Attentional load associated with performing and stabilizing preferred bimanual patterns. J Exp Psychol Human. 1999;25: 1579–1594.

[10] Yogev-Seligmann G, Rotem-Galili Y, Mirelman A, Dickstein R, Giladi N, Hausdorff JM. How does explicit prioritization alter walking during dual-task performance? Effects of age and sex on gait speed and variability. Phys Ther. 2010;90: 177–186. 10.2522/ptj.20090043 20023000 PMC2816029

[11] Zanone P, Monno A, Temprado J, Laurent M. Shared dynamics of attentional cost and pattern stability. Hum Movement Sci. 2001;20: 765–789. 10.1016/s0167-9457(01)00055-0 11792439

[12] McIsaac TL, Benjapalakorn B. Allocation of attention and dual-task effects on upper and lower limb task performance in healthy young adults. 2015;233: 2607–2617. 10.1007/s00221-015-4333-6 26080755

[13] Patel P, Lamar M, Bhatt T. Effect of type of cognitive task and walking speed on cognitive-motor interference during dual-task walking. Neuroscience. 2014;260: 140–148. 10.1016/j.neuroscience.2013.12.016 24345478

[14] Doumas M, Krampe RT. Ecological Relevance Determines Task Priority in Older Adults' Multitasking. J Gerontol B Psychol Sci Soc Sci. 2015;70: 377–385. 10.1093/geronb/gbt105 24149518

[15] Lorist MM, Kernell D, Meijman TF, Zijdewind I. Motor fatigue and cognitive task performance in humans. J Physiol. 2002;545: 313–319. 12433971 10.1113/jphysiol.2002.027938 PMC2290666

[16] Voelcker-Rehage C, Stronge AJ, Alberts JL. Age-related differences in working memory and force control under dual-task conditions. Aging Neuropysiol C. 2006;13: 366–384. 10.1080/138255890969339 16887779

[17] Guillery E, Mouraux A, Thonnard J. Cognitive-Motor Interference While Grasping, Lifting and Holding Objects. 2013. 10.1371/journal.pone.0080125 PMC3820537 24244626

[18] Dettmers C, Lemon RN, Stephen KM, Fink GR, Frackowiak RS. Cerebral activation during the exertion of sustained static force in man. Neuroreport. 1996;7: 2103–2110. 8930968 10.1097/00001756-199609020-00008

[19] Temprado J, Zanone P, Monno A, Laurent M. A dynamical framework to understand performance trade-offs and interference in dual tasks. J Exp Psychol Human. 2001;27: 1303–1313. 11766926

[20] Hiraga CY, Garry MI, Carson RG, Summers JJ. Dual-task interference: attentional and neurophysiological influences. Behav Brain Res. 2009;205: 10–18. 10.1016/j.bbr.2009.07.019 19631693

[21] Richman JS, Moorman JR. Physiological time-series analysis using approximate entropy and sample entropy. Am J Physiol Heart Circ Physiol. 2000;278: H2039–H2049. 10843903 10.1152/ajpheart.2000.278.6.H2039

[22] Donker SF, Roerdink M, Greven AJ, Beek PJ. Regularity of center-of-pressure trajectories depends on the amount of attention invested in postural control. Exp Brain Res. 2007;181: 1–11. 17401553 10.1007/s00221-007-0905-4 PMC1914290

[23] Kang HG, Costa MD, Priplata AA, Starobinets OV, Goldberger AL, Peng CK, et al Frailty and the degradation of complex balance dynamics during a dual-task protocol. J Gerontol A Biol Sci Med Sci. 2009;64: 1304–1311. 10.1093/gerona/glp113 19679739 PMC2781784

[24] Stins J, Michielsen M, Roerdink M, Beek P. Sway regularity reflects attentional involvement in postural control: Effects of expertise, vision and cognition. Gait Posture. 2009;30: 106–109. 10.1016/j.gaitpost.2009.04.001 19411174

[25] Pincus SM, Goldberger AL. Physiological time-series analysis: what does regularity quantify? Am J Physiol. 1994;266: H1643–H1656. 8184944 10.1152/ajpheart.1994.266.4.H1643

[26] Baldissera F, Cavallari P, Civaschi P. Preferential coupling between voluntary movements of ipsilateral limbs. Neurosci Lett. 1982;34: 95–100. 7162702 10.1016/0304-3940(82)90098-2

[27] Baldissera F, Cavallari P, Marini G, Tassone G. Differential control of in-phase and anti-phase coupling of rhythmic movements of ipsilateral hand and foot. Exp Brain Res. 1991: 375–380. 2022245 10.1007/BF00231161

